# Greater widespread functional connectivity of the caudate in older adults who practice kripalu yoga and vipassana meditation than in controls

**DOI:** 10.3389/fnhum.2015.00137

**Published:** 2015-03-16

**Authors:** Tim Gard, Maxime Taquet, Rohan Dixit, Britta K. Hölzel, Bradford C. Dickerson, Sara W. Lazar

**Affiliations:** ^1^Department of Psychiatry, Massachusetts General Hospital, Harvard Medical School, Charlestown, MAUSA; ^2^Bender Institute of Neuroimaging, Justus Liebig Universität Giessen, GiessenGermany; ^3^Faculty of Psychology and Neuroscience, Maastricht University, MaastrichtNetherlands; ^4^Institute of Information and Communication Electronics and Applied Mathematics Institute, Université catholique de Louvain, Louvain-La-NeuveBelgium; ^5^BrainBot, San Francisco, CAUSA; ^6^Department of Neuroradiology, Klinikum Rechts der Isar, Technical University of MunichGermany

**Keywords:** caudate, functional connectivity, graph theory, degree centrality, yoga, mindfulness meditation, aging, basal ganglia-thalamocortical circuits

## Abstract

There has been a growing interest in understanding how contemplative practices affect brain functional organization. However, most studies have restricted their exploration to predefined networks. Furthermore, scientific comparisons of different contemplative traditions are largely lacking. Here we explored differences in whole brain resting state functional connectivity between experienced yoga practitioners, experienced meditators, and matched controls. Analyses were repeated in an independent sample of experienced meditators and matched controls. Analyses utilizing Network-Based Statistics (Zalesky et al., 2010) revealed difference components for yoga practitioners > controls and meditators > controls in which the right caudate was a central node. Follow up analyses revealed that yoga practitioners and meditators had significantly greater degree centrality in the caudate than controls. This greater degree centrality was not driven by single connections but by greater connectivity between the caudate and numerous brain regions. Findings of greater caudate connectivity in meditators than in controls was replicated in an independent dataset. These findings suggest that yoga and meditation practitioners have stronger functional connectivity within basal ganglia cortico-thalamic feedback loops than non-practitioners. Although we could not provide evidence for its mechanistic role, this greater connectivity might be related to the often reported effects of meditation and yoga on behavioral flexibility, mental health, and well-being.

## Introduction

There is a growing interest in the neural correlates of meditation practice. While initial studies focused on the meditative state or the effects of meditation on brain activation during a specific task, more recent studies also have investigated the effect of ongoing regular meditation experience on the resting state of the brain (Jang et al., 2010; Brewer et al., 2011; Kilpatrick et al., 2011; Taylor et al., 2012). These studies have provided first insights in how meditation affects functional brain connectivity at rest. An important limitation of these studies is that they only investigate differences in connectivity between nodes of the default mode network (Buckner et al., 2008) without accounting for the complex network structure that these connections underpin. Recent models of the brain as a complex network has furthered the understanding of its resting state and provided robust methods to compare its properties amongst subjects based on graph theory. These methods refrain from comparing the fMRI signal at every voxel, thereby increasing the statistical power of group comparisons. Therefore this approach is particularly useful for studying the brain resting state between groups of healthy subjects, for which differences may be subtle.

Further, the above-mentioned studies focused only on practitioners of meditation. There is much theoretical debate about how various contemplative practices may be similar or different, both in terms of mechanisms and effects. There is a growing interest in understanding how different contemplative practices compare (Brewer et al., 2011). A study directly comparing different practices may therefore provide invaluable insights into the neural processes involved and provide concrete evidence as to how these practices differ, or not.

In a recent, *hypothesis driven* study, we addressed these issues, and investigated *global* resting state brain functional network properties of yoga- and meditation practitioners (Gard et al., 2014b). Here we use *explorative* methods on the same dataset to investigate *local* differences in the brain resting state functional networks of individuals with extensive meditation or yoga practice compared to demographically matched controls. Unlike previous studies, we use a data-driven approach to reliably identify the differences in networks between the groups across the entire brain, without limiting ourselves to any a priori sub-network or region and without the need of a specific hypothesis. To strengthen confidence in the main finding, we repeated analyses with a second, independent dataset of experienced meditators and controls. Results will be discussed in the light of recent research on the role of the basal ganglia.

## Materials and Methods

### Participants

The first study consisted of 47 participants: 16 yoga practitioners, 16 meditation practitioners, and 15 controls. The three groups were matched for age, sex, education, and handedness. Yoga practitioners were primarily trained in the Kripalu Yoga (Faulds, 2005) tradition and had an average of 13,534 (SD = 9,950) hours of yoga experience. Meditators were primarily trained in Vipassana (a.k.a. insight or mindfulness) meditation (Goldstein and Kornfield, 2001) and had an average of 7,458 h (SD = 5,734) of meditation experience. Controls had less than 4 yoga or meditation classes in the past year and less than 10 classes in their lifetime. See **Table 1** for the demographic characteristics of each group. Participants provided written informed consent and were compensated $100 for their time. The study was approved by the Partners Human Research Committee, Massachusetts General Hospital (protocol 2005P001392). Other data from these subjects has been published elsewhere (Gard et al., 2014b).

**Table 1:** Comparison of demographic variables between controls, yoga practitioners, and meditators for the original dataset.

	Controls		Yoga practitioners		Meditators *M / %*		ANOVA /*χ^2^*-test		
	*M /*%	SD	*M /*%	SD	*M* /*%*	SD	*F /χ^2^*	*df*	*p*
Age (years)	52.93	9.84	49.38	7.79	54.06	8.15	1.29	2, 44	0.286
Education (years)	17.27	1.98	17.31	2.41	18.44	2.58	1.26	2, 44	0.293
Gender (% female)	60%		69%		63%		0.28	2	0.871
Handedness (% right)	87%		88%		88%		0.01	2	0.997

For the replication study we used data of a subset of individuals who participated in a previously published study (Lazar et al., 2005). Resting state BOLD data was available for 13 Vipassana meditation practitioners and 16 controls with little or no meditation experience (less than 4 classes in the past year and less than 10 classes in their lifetime). Meditators had an average experience of 4,831 (SD = 3,738) hours. See **Table 2** for the demographic characteristics of each group. Participants provided written informed consent and were compensated $100 for their time. The study was approved by the Partners Human Research Committee, Massachusetts General Hospital (protocol 2000p-001392).

**Table 2:** Comparison of demographic variables between controls, and meditators for the replication dataset.

	Controls		Meditators *M / %*		*t*-test /*χ^2^*-test		
	*M /*%	SD	*M /*%	SD	*t /χ^2^*	*df*	*p*
Age (years)	36.00	7.67	38.15	7.85	0.74	27	0.463
Education (years)	17.13	1.77	17.54	1.85	0.59	26	0.559
Gender (% female)	44%		31%		0.51	1	0.474
Handedness (% right)	100%		100%				

### Image Acquisition

For the original study data was collected on a Siemens 1.5 Tesla Avanto MRI scanner (Erlagen, Germany) at the Martinos Center for Biomedical Imaging. Structural images were acquired using a T1-weighted magnetization prepared rapid acquisition gradient echo (MPRAGE) sequence (128 sagittal slices, slice thickness = 1.33 mm, TR = 2.73 s, TE = 3.39 ms, flip angle = 7°, field of view = 256 mm × 256 mm, matrix = 192 mm × 192 mm). A 5 min functional resting state scan was acquired using a gradient echo T2^∗^-weighted sequence (TR = 2.5 s, TE = 40 ms, FA = 90°, field of view = 320 mm × 320 mm, matrix = 64 mm × 64 mm). Twenty five sagittal slices with 1 mm gap (voxel size: 3.13 mm × 3.13 mm × 5 mm) were acquired inter-leaved.

For the replication study data was collected on a Siemens 1.5 Tesla Sonata MRI scanner (Erlagen, Germany) at the Martinos Center for Biomedical Imaging. Structural images were acquired using a T1-weighted magnetization prepared rapid acquisition gradient echo (MPRAGE) sequence (128 sagittal slices, slice thickness = 1.33 mm, TR = 2.73 s, TE = 3.39 ms, flip angle = 7°, field of view = 256 mm × 256 mm, matrix = 192 mm × 192 mm). A 6.7 min functional resting state scan was acquired using a gradient echo T2^∗^-weighted sequence (TR = 4 s, TE = 40 ms, FA = 90°, field of view = 320 × 320 mm, matrix = 64 × 64 mm). Twenty-five sagittal slices with 1 mm gap (voxel size: 3.13 mm × 3.13 mm × 5 mm) were acquired inter-leaved. Participants of both the original and the replication study were instructed not to meditate during the resting state scan.

### Analysis

#### Demographics

To test if groups were successfully demographically matched for age and education, ANOVAs and independent sample *t*-tests (two-tailed) were conducted for the original and the replication study, respectively. To evaluate comparability on gender and handedness, χ^2^-tests were conducted for both studies.

#### Data Preprocessing

For both studies resting state data were slice time corrected, realigned, coregistered to individual T1-weighted images, normalized, and spatially smoothed with at 5 mm kernel using SPM8^1^ (Wellcome Department of Cognitive Neurology, London, UK). Next, in the original study the first eight volumes of the functional time series were discarded to allow for stabilization of the MR signal. The remaining 112 volumes were further preprocessed using the Connectivity toolbox^2^ (Whitfield-Gabrieli et al., 2010). In the replication study the first five volumes were discarded and the remaining 95 were further processed in the same way as the data from the original study. Mean white matter signal, mean CSF signal, six motion parameters, and the first order motion derivative were regressed out of the data. Finally, the residual time series were band-pass filtered with a window of 0.008–0.09 Hz.

#### Anatomical Parcellation and Time Series Extraction

Resting state scans were parcellated into 116 regions of interest (ROIs; 90 cortical and subcortical, and 26 cerebellar) using the Automated Anatomical Labeling (AAL; Tzourio-Mazoyer et al., 2002) template in the Wake Forest University (WFU) Pickatlas version 2.5 (Maldjian et al., 2003). For each ROI, the average (of all voxels in the ROI) preprocessed time-series was extracted, resulting in a 116 (ROIs) × 112 (volumes) time-series matrix for each subject. Time-series extraction was done with the Connectivity toolbox^3^ (Whitfield-Gabrieli et al., 2010).

#### Network Analysis

Networks are defined as a set of nodes connected by links. In the context of functional network analysis, the nodes are each of the 116 regions of interest and the links represent the strength of the connections between them.

Anatomical parcellation and time series extraction result in a time-series matrix for each subject. The correlations between each pair of time series of each such correlation matrix were computed, resulting in a 116 × 116 correlation matrix. The elements of this matrix are therefore real numbers between -1 and 1. All negative entries were set to zero so that all elements belong to [0,1], which is a necessary step to obtain a network with positive weights (Schwarz and McGonigle, 2011).

These matrices essentially define networks wherein the (i,j) entry of the matrix is the strength of the connection between the i-th and j-th ROI. These networks are weighted (because the connections can have any value between zero and one) and undirected [because the (i,j) entry of the matrix equal the (j,i) entry]. Our choice of using weighted networks instead of unweighted ones (obtained by further binarizing positive weights) is motivated by (Wang et al., 2011) showing that analysis of weighted networks is more reliable and (Barrat et al., 2004) showing that binarization results in a loss of valuable information. The networks were then analyzed using NetworkX (Hagberg et al., 2008).

#### Network-Based Statistics

One-to-one comparisons between groups for each connections in the network would result in many comparisons to be made. These comparisons may lack statistical power due to the need to correct for multiple comparisons. To explore differences in resting state brain functional connectivity between yoga practitioners, meditators, and controls, while considering the entire brain network, we therefore employed Network-Based Statistics (NBS) which detects clusters of connections (instead of individual connections) that significantly differ between group. NBS is a solution to the multiple comparison problem. NBS assumes that edges contributing to population differences tend to appear in connected components (Zalesky et al., 2010). Introducing this assumption decreases the number of comparisons and unveils clusters of edges that significantly differ between the groups.

More specifically, we used the NBS method for the comparisons yoga practitioners > controls, meditators > controls, and yoga practitioners versus meditators (two-sided test). For the comparisons involving controls we used one-sided tests, based on previous studies that found greater resting stage connectivity in meditators compared to controls (Brewer et al., 2011). Much like cluster-based statistics, NBS requires a threshold on the *t*-statistics (or equivalently on the *p*-value) of individual edge differences. Connected components are subsequently defined in the binary network of supra-threshold edges. To explore spatially small, hence interpretable, subnetworks, we used a relatively severe initial threshold of *p* < 0.00005.

#### Degree Centrality

Network-based statistics limits the number of comparisons by automatically and reliably identifying subnetworks of interests. To further investigate the central role that the caudate (central node of the detected subnetworks) plays in the functional networks for yoga practitioners > controls and meditators > controls, degree centrality of the caudate was computed for each subject (Eq. 1). Degree centrality was chosen as it is conceptually the simplest measure of nodal importance in a network. Degree centrality is defined as:

(1) $$C_{D}(v)=\frac{\deg\left(v\right)}{n-1}$$

where deg(*v*) is the weighted degree of the node *v* (i.e., the sum of the strengths of its connections) and n is the total number of nodes in the network. A larger degree centrality therefore implies that the node is more connected to the rest of the network.

We compared the degree centrality of controls, meditators, and yoga practitioners for the left and right caudate nuclei. Since the assumption of homogeneity of variances was not met, we used a Welch’s test to assess the equality of means in the population. The test was followed up by independent two-tailed *t*-tests comparing yoga practitioners, meditators, and controls pairwise. To validate the findings from these analyses, we tested the hypothesis that meditators have greater degree centrality than controls (independent samples *t*-test, one-tailed) in an independent dataset of 13 meditators and 16 controls.

#### Individual Edges

To follow up the finding of greater degree centrality of the caudate in yoga practitioners and meditators vs. controls, connectivity between the caudate and each of the 115 other nodes in the network was compared between yoga practitioners and controls and meditators and controls. To do so, correlation coefficients were Fisher-transformed (Eq. 2) to obtain normally distributed values which were used for the second-level node-wise analysis.

(2) $$z=\frac{1}{2}\,\;\ln\;\frac{1+r}{1-r}=\mathrm{arctanh}\left(r\right),$$

where *r* represents the correlation coefficient value.

Then, independent samples *t*-tests (two-tailed) were calculated for each edge for yoga practitioners vs. controls, and meditators vs. controls. Correction for multiple comparisons was done using the False Discovery Rate (FDR) <0.5; Genovese et al., 2002). Fisher transformation, and the node-wise analysis was done with the Connectivity toolbox^4^ (Whitfield-Gabrieli et al., 2010). Again, to validate the findings from this analysis we repeated the above analyses on an independent dataset of 13 meditators and 16 controls.

#### Cognitive and Practice Assessment

Due to the role of the caudate in aging and cognitive functioning, the relation between degree centrality in the left and right caudates and age and fluid intelligence was explored. This was done by calculating Pearson product moment correlations within groups and over the merged groups. Fluid intelligence was measured with the odd items of the Raven’s Advanced Progressive Matrices (APM; Raven et al., 1998; Raven, 2000). In addition, the relationship between amount of yoga- or meditation practice and degree centrality in the left and right caudates was explored in the yoga- and meditation groups, using Pearson product moment correlations. Amount of lifetime practice was based on participant’s self-reported estimates.

## Results

To test the success of participant matching in both the original as well as in the replication data set, ANOVAs, *t*-tests and χ^2^-test were conducted. There were no significant differences in age, education, gender, and handedness between yoga practitioners, meditators, and controls in the original data set (**Table 1**). In the replication data set there also were no differences on these variables between meditators and controls (**Table 2**).

### Network-Based Statistics

**FIGURE 1:**
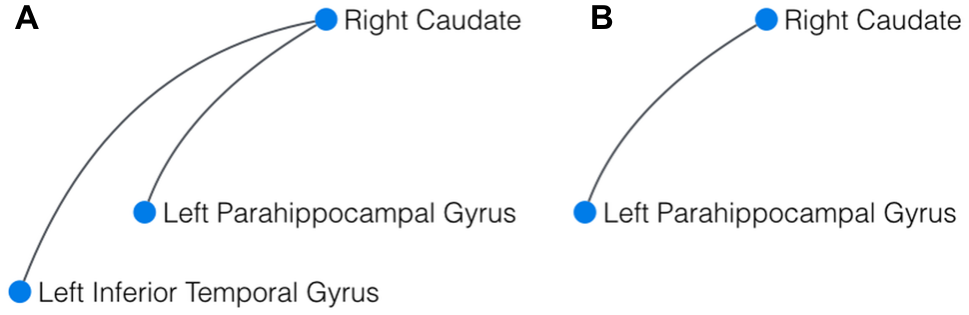
**Result of the network-based statistics (NBS). (A)** In yoga practitioners vs. controls, NBS revealed a significant cluster of three interconnected components, centered around the right caudate. **(B)** In meditators vs controls, the largest component detected by NBS also comprised the right caudate and the left parahippocampal gyrus.

To compare whole brain resting state networks of yoga practitioners, meditators, and controls, the (NBS; Zalesky et al., 2010), a novel approach to correct edgewise connectivity for multiple comparisons, was used. At the stringent initial p-threshold of *p* < 0.00005, this approach revealed a significant (*p* = 0.031) difference component for the comparison yoga practitioners > controls. This component was comprised of three nodes and two edges, with the right caudate serving as the central node, connected to the left parahippocampal gyrus and the left inferior temporal gyrus (**Figure 1A**). The comparisons meditators > controls and yoga practitioners versus meditators did not reveal significant difference components. Although not significant (*p* = 0.176), it is striking to note that the largest difference component for meditators > controls was comprised of the same two connected nodes, namely the right caudate and the left parahippocampal gyrus as in the difference network for yoga practitioners > controls (**Figure 1B**).

### Degree Centrality Caudate

**FIGURE 2:**
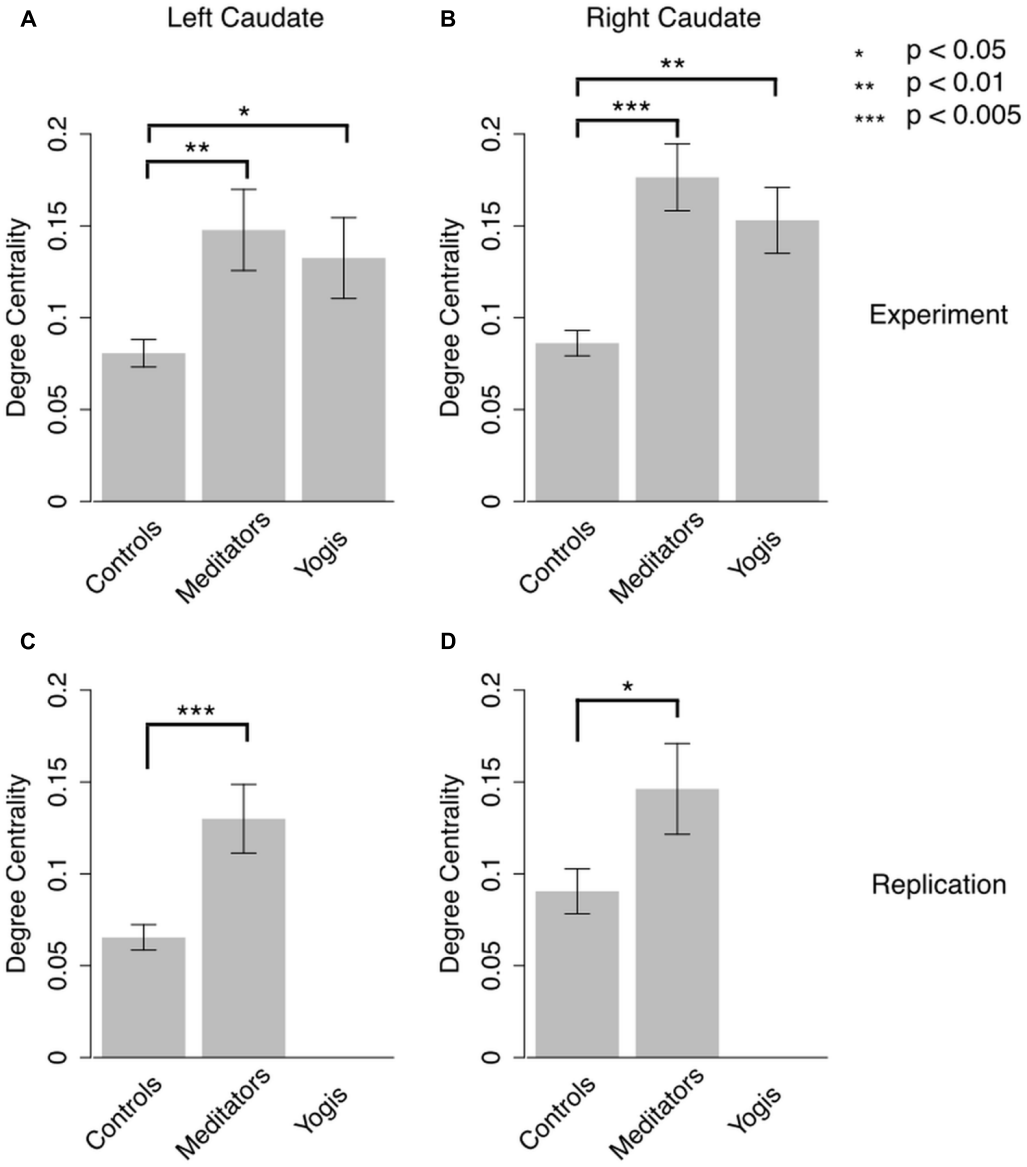
**Degree centrality in left and right caudate in the original experiment **(A,B)** and in the replication study **(C,D)**.** Error bars represent SE of the mean. *P*-values are based on independent samples *t*-tests.

To further investigate the central role of the caudate in the identified components, we calculated the degree centrality of the right and left caudate for each participant’s weighted network and compared it between groups. Welch’s test of equality of means, which is an alternative to ANOVA when the assumption of homogeneity of variances is not met (Welch, 1951), revealed that the mean degree centrality was different for yoga practitioners, meditators, and controls in the right [*F*(2,24.576) = 14.587, *p* < 0.001] and in the left caudate [*F*(2,23.785) = 5.867, *p* = 0.008]. *Post hoc* independent samples *t*-tests (two-tailed) revealed that this effect in the left caudate was driven by greater weighted degree in yoga practitioners [*M* = 0.132, SD = 0.088; *t*(18.390) = 2.171, *p* = 0.038] and meditators [*M* = 0.148, SD = 0.088; *t*(19.362) = 2.801, *p* = 0.009] than in controls (*M* = 0.081, SD = 0.029; **Figure 2A**). There was no significant difference between yoga practitioners and meditators [*t*(30) = 0.490, *p* = 0.628]. The effect in the right caudate also was driven by greater weighted degree in yoga practitioners [*M* = 0.153, SD = 0.072; *t*(19.318) = 3.472, *p* = 0.003] and meditators [*M* = 0.176, SD = 0.073; *t*(19.224) = 4.641, *p* < 0.001] than in controls (*M* = 0.086, SD = 0.027; **Figure 2B**). There was no significant difference between yoga practitioners and meditators [*t*(30) = 0.916, *p* = 0.367].

In the collapsed sample degree centrality of the left and right caudates were not significantly correlated with age [*r*(45) = 0.033, *p* = 0.823 and *r*(45) = -0.64, *p* = 0.671, respectively] or fluid intelligence [*r*(45) = 0.130, *p* = 0.385, and *r*(45) = 0.043, *p* = 0.776, respectively]. Also in controls degree centrality of the left and right caudates were not significantly correlated with age [*r*(13) = -0.089, *p* = 0.753, and *r*(13) = -0.097, *p* = 0.731, respectively] or fluid intelligence [*r*(13) = 0.057, *p* = 0.839, and *r*(13) = -0.050, *p* = 0.861, respectively]. Similarly in meditators degree centrality of the left and right caudates were not significantly correlated with age [*r*(14) = -0.202, *p* = 0.453, and *r*(14) = -0.248, *p* = 0.354, respectively], fluid intelligence [*r*(14) = 0.063, *p* = 0.817, and *r*(14) = 0.183, *p* = 0.499, respectively], or meditation practice [*r*(11) = -0.051, *p* = 0.868, and *r*(11) = -0.279, *p* = 0.355, respectively]. In yoga practitioners degree centrality of the left and right caudates were also not significantly correlated with age [*r*(14) = 0.070, *p* = 0.798, and *r*(14) = 0.345, *p* = 0.191, respectively], fluid intelligence [*r*(14) = -0.353, *p* = 0.181, and *r*(14) = -0.314, *p* = 0.236, respectively], or yoga practice [*r*(11) = 0.292, *p* = 0.291, and *r*(11) = 0.418, *p* = 0.121, respectively].

These findings were replicated in an independent dataset of 13 meditators and 16 controls. Meditators (*M* = 0.130, SD = 0.067) had significantly greater degree centrality in the left caudate than controls [*M* = 0.065, SD = 0.028; *t*(15.269) = 3.238, *p* = 0.003; **Figure 2C**]. In the right caudate meditators (*M* = 1.463, SD = 0.089) also had greater degree centrality than controls [*M* = 0.090, SD = 0.049; *t*(17.746) = 2.021, *p* = 0.028; **Figure 2D**]. Degree centrality in the left and right caudates were not significantly correlated with age in meditators, [*r*(14) = -0.018, *p* = 0.954, and *r*(14) = 0.102, *p* = 0.741, respectively], controls [*r*(14) = 0.220, *p* = 0.414, and *r*(14) = -0.056, *p* = 0.837, respectively], or in the collapsed sample [*r*(27) = 0.126, *p* = 0.516, and *r*(27) = 0.088, *p* = 0.651, respectively]. Amount of meditation practice was significantly correlated with degree centrality in left [*r*(11) = -0.604, *p* = 0.029] but not right [*r*(11) = 0.124, *p* = 0.687] caudate.

### Individual Edges

**FIGURE 3:**
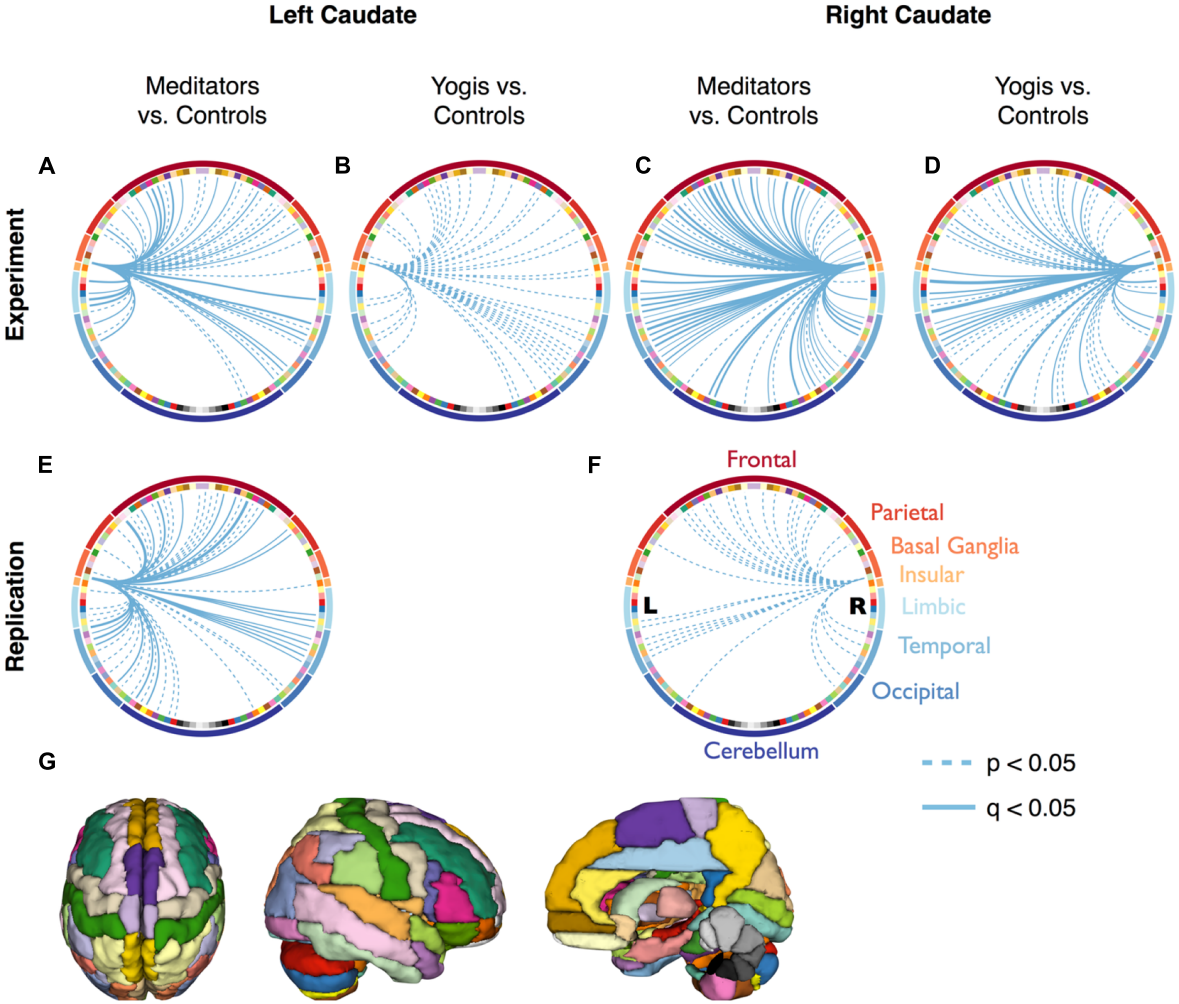
**Connectivity between the caudate and all 115 other regions of the Automated Anatomical Labeling (AAL) atlas.** Continuous lines represent significantly stronger connections compared to controls, after False Discovery Rate (FDR) correction (*q* < 0.05). Dashed lines represent significantly stronger connections without FDR correction (*p* < 0.05). **(A–D)** are based on the original dataset, while **(E,F)** are based on the replication data-set. The colors of the nodes in **(A–F)** correspond to the parcellation in **(G)** and are clustered according to the main brain region they belong to (frontal, parietal, limbic, temporal, and occipital lobes, and basal ganglia, insular cortex, and cerebellum). Significant connections and their *p*-values are listed in Supplementary Tables S1–S3.

To find out by what edges the greater centrality in yoga practitioners and meditators as compared to controls was driven, the average connectivity between the caudate and all 115 other brain regions was compared pairwise between yoga practitioners, meditators and controls. Yoga practitioners and meditators had stronger connectivity to a large number of brain regions as compared to controls, while there were no differences between yoga practitioners and meditators (Figures 3A–D,G, Tables S1–S2). The finding of greater connectivity between the caudate and a large number of brain regions in meditators than in controls was replicated in the independent dataset (Figures 3E–G, Table S3).

## Discussion

Here we explored differences in resting state functional networks between yoga practitioners, meditators, and controls. Our data revealed that the caudate was a hub in the difference network (i.e., the network whose edges represent the differences between the groups) between yoga practitioners and controls. Not only was the caudate a hub in the difference network, yoga practitioners and meditators also had greater degree centrality in the caudate than controls. Further *post hoc* analyses revealed this greater degree centrality was not driven by a specific connection but rather by widespread stronger connectivity between the caudate and multiple regions across the rest of the brain.

### Similarities between Yoga and Mindfulness Meditation

This finding of widespread stronger connectivity of the caudate in both yoga practitioners and meditators is consistent with overlapping theoretical mechanisms involved in both practices. Both Kripalu Yoga and Vipassana theoretically and empirically foster mindfulness, thereby sharing a key aspect (Faulds, 2005; Chiesa, 2010; Gard et al., 2012a; Perelman et al., 2012). This overlap between the practices might also be the reason why we did not find significant differences in global (Gard et al., 2014b) and local (this study) resting state brain networks between yoga practitioners and meditators. With a larger sample size it might be possible to identify more subtle differences in resting state network organization between yoga practitioners and meditators. It will also be interesting to test contemplative traditions that do not emphasize mindfulness.

### The Caudate Findings in Yoga and Meditation

This is, to our knowledge, the first study revealing greater resting state functional connectivity between the caudate and a variety of brain regions in experienced yoga practitioners and meditators as compared to controls. Our finding of greater degree centrality in the caudate of meditators is particularly strong as it was replicated in an independent sample, collected at a different time, on a different scanner and with different scanning parameters. Remarkably, despite these technological differences, not only the statistical significance of the differences but also the magnitude of these differences were replicated (**Figure 2**).

The correlation between degree centrality in the left caudate and amount of meditation practice in the replication sample suggests that functional connectivity of the caudate is related to meditation experience. The negative direction of this relation in combination with greater caudate connectivity in meditators than in controls resembles the pattern of an inverted u-shape that has previously been reported in meditators with regard to brain activity (e.g., Brefczynski-Lewis et al., 2007; Gard et al., 2012b). However, in the original sample no relation between amount of practice and degree centrality of the caudate was found. This might be the result of a small sample size and the fact that imprecise practice estimates, as participants indicated that it was difficult to recall their lifetime practice. Structural findings.

Although not much is known about the role of the caudate in meditation, this structure has been reported in several meditation-related brain imaging studies. Structural brain imaging studies revealed increased gray matter volume/density in the caudate after completion of 8-week mindfulness-based interventions (Farb et al., 2013; Pickut et al., 2013), while dispositional mindfulness in non-meditators has been reported to be negatively correlated with caudate volume (Taren et al., 2013).

### Functional Activation Findings

Functional studies have revealed increased activity in this region during the state of meditation. Lazar et al. (2000) for example found increased caudate activation during kundalini silent mantra meditation as compared to a silent random word generation task in experienced kundalini meditation practitioners. Dickenson et al. (2013) reported increased caudate activity in novice mediators during mindful breath awareness as compared to mind wandering. Another study that assessed activity during different phases in the interplay between mindful awareness and mind wandering in moderately experienced meditators revealed increased caudate activity during the attention shifting phase, when attention was shifted to the breath, as compared to mind wandering phase (Hasenkamp et al., 2012). Brefczynski-Lewis et al. (2007) also investigated different phases of the meditation process but in Tibetan Buddhist meditators with different levels of experience. They reported increased caudate activity only during the startup phase (first 10 s) of a concentrative meditation in the most experienced meditators (>37,000 h experience), while the less experienced long term meditators (10,000–24,000 h) also had increased caudate activity during the continuation phase of the meditation session. Another study that included a mix of Tibetan Buddhist and Zen meditators reported increased caudate activation during continuous meditation as compared to rest, although the analysis was not corrected for multiple comparisons (Baerentsen et al., 2009). The involvement of the caudate in the meditative state as compared to rest has further been confirmed by a meta analysis that included studies with a wide variety of meditation practices (Sperduti et al., 2012).

Despite clear differences on the surface, different meditation techniques seem to have some overlap in their neural basis, including in the caudate. However, one study investigating experienced Kria Yoga practitioners reported decreased activation of caudate during meditation (guided imagery) as compared to rest (Lou et al., 1999). This discrepancy might be due to that specific meditation practice, or to the fact that just before the experiment in the scanner started, participants had practiced an intense form of concentrative meditation for 2 h. Methodological differences between this early and the more recent studies may be another reason.

Increased caudate activity has also been reported at rest in novices after completing a short integrative body-mind meditation training (Tang et al., 2009). Furthermore, a sample of experienced meditators from a variety of traditions, including Tibetan Buddhist meditators ,and Franciscan nuns, have been shown to have greater caudate activity at rest than matched controls (Newberg et al., 2010).

### Functional Connectivity Findings

The state of meditation has also been investigated in terms of brain connectivity. Baerentsen et al. (2009) performed Independent Component Analysis (ICAs) on the fMRI time-series during sustained meditation. This analysis revealed a number of components including one large component comprising the caudate, the lateral prefrontal cortex, the precentral gyrus, the insula, the temporal gyrus, the parahippocampal gyrus, the fusiform gyrus, the ligual gyrus, and the cerebellum. Our findings are aligned with this finding of a meditation-related network that involves the caudate, alongside frontal, and temporal brain regions. We extend the finding by revealing this network in both meditators and yoga practitioners, at rest, and by revealing the central role of the caudate in this network.

### Basal Ganglia-Thalamocortical Loops

Most studies so far have only reported meditation-related caudate activity as a side finding and have not attempted to interpret it extensively. However, the role of the caudate in meditation has been discussed in a number of theoretical accounts. In these theories the caudate is discussed as a key component of the basal ganglia-thalamocortical circuits. These segregated circuits originate in functionally related cortical regions that send excitatory glutamatergic projections to specific parts of the striatum, which then send converging projections to the pallidus and substantia nigra through a direct net-inhibitory and an indirect net-excitatory pathway. Both pathways to the basal ganglia output regions are mostly GABAergic and are modulated by dopaminergic projections from the midbrain, resulting in net inhibition of the neurons in the output regions. The latter have GABAeric projections to specific thalamic nuclei which project back (glutamatergic) to the main prefrontal area that fed the loop and after which the loop is named. In three out of the five known loops, the oculomotor-, dorsolateral prefrontal-, and lateral orbitofrontal loops, the caudate is the central striatal component (Alexander et al., 1986; Alexander and Crutcher, 1990). In the oculomotor loop the caudate receives input from the frontal eyefields, the dorsolateral prefrontal cortex, and the posterior parietal cortex, and in the dorsolateral prefrontal loop from the posterior parietal cortex and the arcuate premotor area. In the lateral orbitofrontal loop it receives input from the superior and inferior temporal gyrus and from the anterior cingulate cortex (Alexander et al., 1986).

These frontal–subcortical loops have been related to a variety of human behaviors, including alterations in emotion and cognition as a result of lesions (Cummings, 1993). As Graybiel (2000) noted “Under conditions of circuit dysfunction, at one extreme excessive and repetitive actions or thoughts could result, and at the other extreme poverty of movement or thought could be the result.” A recent meta-analysis has related caudate functional connectivity in particular to cognition, emotion, action, and perception (Robinson et al., 2012).

With its broad converging cortical input, its gating function on the thalamus, and its modulation by the dopaminergic reward system, the basal ganglia are implicated in reinforcement learning: learning to take actions that maximize reward (Braunlich and Seger, 2013). Two types of reinforcement learning can be distinguished: model-based or goal-directed and model-free or habitual learning. The former involves value based and contingency learning and results in behavioral flexibility. The latter involves simple stimulus response learning and although low in computational cost it is not adaptive in changing environments (Schwabe and Wolf, 2011; Doll et al., 2012; Braunlich and Seger, 2013). While both types of learning utilize dopamine mediated reward prediction error signaling from the ventral tegmental area and the substantia nigra (Schultz et al., 1997), goal directed learning is mediated by the caudate and habitual learning by the putamen (Braunlich and Seger, 2013). Indeed, a recent study revealed that flexible goal-directed behavior was predicted by white matter structural connectivity between caudate and ventromedial prefrontal cortex while non-adaptive habitual behavior was predicted by connectivity between the putamen and the premotor cortex (de Wit et al., 2012). This finding combined with our finding of greater widespread caudate connectivity in yoga practitioners and meditators might suggest that the positive association between mindfulness and cognitive and behavioral flexibility (Carmody et al., 2009; Anicha et al., 2012) is mediated by connectivity between caudate and prefrontal cortex. Interestingly, stress which can be reduced through meditation and yoga (Carmody and Baer, 2008; Gard et al., 2012a), has been shown to result in a shift from goal-directed to habitual behavior (Schwabe and Wolf, 2011).

### Theoretical Models of Yoga and Meditation

Some theoretical models of meditation and yoga incorporate basal ganglia–thalamocortical circuits. In the model of Vago and Silbersweig (2012) for example, these loops are closely related to the experiential enactive self (EES) network, one of four networks that they hypothesize to underlie self-awareness, self-regulation, and self-transcendence through mindfulness. The EES refers to a non-conscious sensory-affective-motor learning network that Vago and Silbersweig (2012) hypothesized to support attention regulation and awareness of sensory and mental activity. In a more recent paper, Vago (2014) explicitly extends this view to habits of minds. Similarly Gard et al. (2014a) have proposed that yoga practice also involves basal ganglia cortico-thalamic circuits that are involved in extinction learning to unlearn old, maladaptive behavioral patterns, and to establish new, adaptive ones.

Travis and Wallace (1999) proposed that the state of meditation is initiated by a “neural switch” network involving frontal brain regions and is further maintained by a “maintenance” network that involves basal ganglia cortico-thalamic feedback loops. Similarly Newberg and Iversen (2003) in their neurochemical model of meditation proposed that meditation is initiated by frontal brain regions and maintained by basal ganglia cortico-thalamic feedback loops. The involvement of these frontal-subcortical loops in this model is supported by the finding of increased dopamine release in the striatum during the yoga nidra meditation (Kjaer et al., 2002) and increased GABA levels in the thalamus after yoga practice (Streeter et al., 2007, 2010). However, in contrast to these models, a recent fMRI study (Baerentsen et al., 2009) did not find evidence for the frontal involvement but rather increased brain activity in the putamen at the onset of meditation. During sustained meditation, increased activation in the caudate was reported.

Based on these findings, the previously proposed models and their own meta-analysis, Sperduti et al. (2012) proposed a three component model for the state of meditation comprising an “interference control system,” a “thoughts monitoring system,” and a “self monitoring system.” It is the interference control system that would support both the switching to and the maintenance of the meditative state through involvement of the putamen and the caudate as part of a basal ganglia cortico-thalamic feedback system. Sperduti et al. (2012) note that their model is based on increased brain activation during meditation as compared to baseline and suggest that it should be further validated with other methods including functional connectivity.

The fact that greater connectivity was not driven by single strong connections but by wide-spread connections including those to frontal, temporal and parietal regions, further suggests that yoga practitioners and meditators have more efficient basal ganglia cortico-thalamic feedback loops than controls. This enhanced basal ganglia cortico-thalamic feedback loop functioning even during a state of rest might be the result of repeated involvement of these loops during the state of meditation as proposed in the models of Travis and Wallace (1999), Newberg and Iversen (2003), Sperduti et al. (2012), Vago and Silbersweig (2012), and Gard et al. (2014a). This lasting change in basal ganglia cortico-thalamic feedback loops might be of clinical relevance as disturbances in these loops have been associated with mental health disorders, e.g., autism (Turner et al., 2006), obsessive compulsive disorder (Harrison et al., 2009), schizophrenia (Salvador et al., 2010; Simpson et al., 2010), and depression (Bluhm et al., 2009). This combined with our finding of increased connectivity between caudate and several brain regions, and the fact that mindfulness based interventions have been shown to improve mental health (Grossman et al., 2004), leads to the hypothesis that improved mental health due to mindfulness may be mediated in part by connectivity in caudate.

### Aging, Cognition, and Caudate Connectivity

Normal aging and mild cognitive impairment are also both associated with decreased caudate connectivity (Klostermann et al., 2012; Morbelli et al., 2012; Podell et al., 2012; Agosta et al., 2013, but see Tomasi and Volkow, 2012), and caudate dopamine D1 receptor density (Rieckmann et al., 2011). Although we did not find a significant correlation between age and degree centrality in the caudate in the current samples, the greater degree centrality in caudate in yoga and meditation practitioners may be the result of decreased age-related decline rather than an increased caudate connectivity due to practice. Interestingly, studies have shown that the greater caudate-frontal connectivity in younger subjects is associated with better working memory performance (Klostermann et al., 2012; Podell et al., 2012) and that the shape and volume of the striatum are related to intelligence (Burgaleta et al., 2014; MacDonald et al., 2014). Based on these findings, combined with our previous finding in the current sample that age-related decline in fluid intelligence is offset in yoga and meditation practitioners (Gard et al., 2014b), it might be hypothesized that decreased age-related decline in fluid intelligence in yoga and meditation practitioners is mediated in part by increased caudate connectivity. However, we did not find a significant correlation between fluid intelligence and degree centrality in caudate, possibly due to power limitations.

### Limitations

This study has several limitations. The design is cross sectional so no inference about the causality of the greater caudate connectivity can be made. Furthermore, although instructed to rest and not to meditate, there is no objective way to verify that participants were not actively meditating during the resting state scan. Lastly, as participants in the first sample as well as half of the participants in the replication study are of middle age, it is not clear if greater caudate connectivity is the result of a meditation-related increase or an offset of age-related decline. This limitation of potential age-related effects is a problem in many studies with experienced yoga and meditation practitioners as many practitioners with extensive practice tend to be older.

## Conclusion

In summary, we have demonstrated in two independent datasets that yoga practitioners and meditators have greater degree centrality in the caudate than matched controls. *Post hoc* analyses of both datasets revealed that the greater connectivity of the caudate was driven by wide spread connectivity to most of the cerebrum, including frontal, temporal, and parietal brain regions.

These findings provide evidence for the previously hypothesized involvement of basal ganglia cortico-thalamic feedback loops in meditation (Sperduti et al., 2012) and yoga (Gard et al., 2014a). At the same time they extend these hypotheses by revealing stronger caudate connectivity not only in meditators but also in yoga practitioners. There have been relatively few neuroimaging studies of yoga practitioners and no studies directly comparing yoga and meditation practitioners, thus these data provide important information suggesting that different contemplative practices may have some similarities at the neural level. The current study was relatively small and was cross-sectional in design, so further studies with larger sample sizes and longitudinal designs are necessary to reveal the more subtle differences between the two. Our findings also extend previous hypotheses involvement of basal ganglia cortico-thalamic feedback loops during a state of meditation by providing support for increased connectivity in these loops during rest. This increased connectivity in these loops could be a potential mechanism accounting for improved behavioral flexibility, mental health, and well-being that is associated with yoga and meditation. Further research is required to test this hypothesis.

## Conflict of Interest Statement

The first study was partially funded by the Kripalu Institute for Extraordinary Living. As the study is in part about yoga, this might be perceived as a conflict of interest. However, the Kripalu Institute for Extraordinary Living was not involved in data collection, analysis, and manuscript writing at all.

## Footnotes

1 http://www.fil.ion.ucl.ac.uk/spm/

2 http://www.nitrc.org/projects/conn

3 http://www.nitrc.org/projects/conn

4 http://www.nitrc.org/projects/conn
