# Supplementary material for: Greater widespread functional connectivity of the caudate in older adults who practice kripalu yoga and vipassana meditation than in controls
Source: Front Hum Neurosci. 2015 Mar 16;9:137. doi: 10.3389/fnhum.2015.00137 (PMC4360708; doi:10.3389/fnhum.2015.00137)
Supplement: Supplementary file 1 [file data_sheet_1.pdf]

Supplementary Material

Greater widespread functional connectivity of the caudate in older adults who practice kripalu yoga and vipassana meditation than in controls.

Tim Gard[1,2,3†*], Maxime Taquet[4†], Rohan Dixit[5], Britta K. Hölzel[1,6], Bradford C. Dickerson[1], Sara W. Lazar[1]

[1]Massachusetts General Hospital, Harvard Medical School, Charlestown, MA, USA
[2]Bender Institute of Neuroimaging, Justus Liebig Universität Giessen, Giessen, Germany
[3]Faculty of Psychology and Neuroscience, Maastricht University, Maastricht, The Netherlands
[4]ICTEAM Institute, Université catholique de Louvain, Louvain-La-Neuve, Belgium
[5]BrainBot, San Francisco, CA, USA
[6]Department of Neuroradiology, Klinikum rechts der Isar, TechnicalUniversity of Munich, Germany
[†]Authors contributed equally

***Correspondence:** Tim Gard, Massachusetts General Hospital, Department of Psychiatry, 120 2nd Ave, Charlestown, MA 02129, USA|
tgard@nmr.mgh.harvard.edu

Supplementary Tables

Table S1. P-values of significant connections between the left caudate and all 115 other regions of the AAL atlas for meditators vs controls and yogis vs controls in the experimental data-set, as displayed in Figure 3a and 3b.

Experiment - Left caudate					
Meditators vs Controls (Figre 3a)			Yogis vs Controls (Figure 3b)		
Node	Hemisphere	*p*	Node	Hemisphere	*p*
CingulumMid	Left	0.00037	CingulumMid	Left	0.00183
CingulumAnt	Left	0.00060	TemporalMid	Right	0.00220
ParaHippocampal	Left	0.00087	FrontalInfTri	Left	0.00278
FrontalInfTri	Left	0.00096	TemporalSup	Right	0.00303
CingulumMid	Right	0.00097	Precentral	Right	0.00321
TemporalMid	Right	0.00098	Cuneus	Right	0.00441
SuppMotorArea	Left	0.00166	SuppMotorArea	Left	0.00468
FrontalInfOrb	Left	0.00167	CingulumMid	Right	0.00545
Heschl	Left	0.00319	TemporalMid	Left	0.00608
FrontalInfOper	Left	0.00375	Rectus	Left	0.00833
TemporalSup	Right	0.00391	Postcentral	Right	0.00844
Cuneus	Right	0.00423	ParietalInf	Left	0.00917

Table S1. Continued.

Experiment - Left caudate					
Meditators vs Controls (Figre 3a)			Yogis vs Controls (Figure 3b)		
Node	Hemisphere	*p*	Node	Hemisphere	*p*
ParietalInf	Left	0.00504	Heschl	Right	0.01036
Heschl	Right	0.00546	OccipitalSup	Right	0.01040
Precentral	Right	0.00583	ParietalInf	Right	0.01396
FrontalInfOper	Right	0.00584	FrontalInfOrb	Left	0.01473
Lingual	Right	0.00672	FrontalMedOrb	Right	0.01509
TemporalMid	Left	0.00705	Amygdala	Right	0.01566
FrontalSupMedial	Left	0.00807	SupraMarginal	Left	0.01635
CingulumPost	Left	0.00817	CingulumAnt	Left	0.01805
SuppMotorArea	Right	0.00830	CerebelumCrus2	Right	0.01820
Cerebelum10	Right	0.00832	FrontalInfOper	Right	0.01869
FrontalMedOrb	Right	0.00873	Calcarine	Right	0.02133
Precentral	Left	0.00960	Insula	Right	0.02284
ParietalInf	Right	0.01087	Heschl	Left	0.02599
FrontalMid	Left	0.01088	OccipitalMid	Right	0.02677
ParacentralLobule	Right	0.01206	FrontalInfOper	Left	0.02912
FrontalSup	Right	0.01229	Precentral	Left	0.03015
Cerebelum45	Right	0.01315	Putamen	Left	0.03135
RolandicOper	Left	0.01450	Cerebelum7b	Right	0.03267
TemporalInf	Left	0.01731	FrontalSupMedial	Left	0.03410
FrontalMidOrb	Right	0.01864	Lingual	Right	0.03483
ParietalSup	Left	0.01877	TemporalSup	Left	0.03553
Fusiform	Right	0.02082	RolandicOper	Left	0.03945
FrontalSupOrb	Right	0.02343	OccipitalInf	Right	0.04064
Precuneus	Right	0.02557	FrontalMidOrb	Right	0.04260
Cerebelum3	Right	0.02654	Cerebelum3	Right	0.04266
TemporalSup	Left	0.02871	Cerebelum10	Right	0.04273
FrontalSup	Left	0.02909	OccipitalMid	Left	0.04432
Insula	Right	0.02982	SuppMotorArea	Right	0.04535
SupraMarginal	Left	0.03215			
Calcarine	Right	0.03419			
ParacentralLobule	Left	0.03447			
FrontalInfTri	Right	0.03557			
Postcentral	Right	0.04140			
RolandicOper	Right	0.04225			
SupraMarginal	Right	0.04327			
TemporalPoleSup	Right	0.04541			

Table S2. P-values of significant connections between the right caudate and all 115 other regions of the AAL atlas for meditators vs controls and yogis vs controls in the experimental data-set, as displayed in Figure 3c and 3d.

Experiment – Right caudate					
Meditators vs Controls (Figure 3c)			Yogis vs Controls (Figure 3d)		
Node	Hemisphere	*p*	Node	Hemisphere	*p*
FrontalInfOrb	Left	0.00001	ParaHippocampal	Left	0.00007
TemporalPoleSup	Left	0.00023	TemporalInf	Left	0.00008
FrontalSup	Left	0.00031	Cerebelum3	Left	0.00019
Precentral	Left	0.00047	ParaHippocampal	Right	0.00128
Insula	Left	0.00063	Pallidum	Right	0.00193
CingulumMid	Left	0.00068	SuppMotorArea	Left	0.00196
ParietalInf	Left	0.00071	TemporalSup	Left	0.00244
SuppMotorArea	Left	0.00082	CingulumPost	Left	0.00285
SuppMotorArea	Right	0.00083	Heschl	Left	0.00325
FrontalSupMedial	Left	0.00083	TemporalPoleSup	Right	0.00326
Cerebelum3	Left	0.00086	FrontalMedOrb	Right	0.00360
Cerebelum45	Right	0.00095	Insula	Left	0.00451
Fusiform	Left	0.00097	Angular	Left	0.00463
ParacentralLobule	Left	0.00097	FrontalInfOrb	Right	0.00492
FrontalInfTri	Left	0.00101	Cerebelum45	Right	0.00550
TemporalPoleSup	Right	0.00109	FrontalSupMedial	Left	0.00649
ParietalSup	Left	0.00119	Fusiform	Left	0.00666
Cerebelum45	Left	0.00129	Vermis6	-	0.00721
ParaHippocampal	Left	0.00131	FrontalInfOrb	Left	0.00756
FrontalInfOrb	Right	0.00136	TemporalPoleSup	Left	0.00783
CingulumAnt	Left	0.00153	SuppMotorArea	Right	0.00820
Heschl	Left	0.00170	TemporalPoleMid	Right	0.00838
TemporalInf	Left	0.00172	ParietalInf	Left	0.00954
RolandicOper	Left	0.00198	CingulumMid	Left	0.00960
TemporalSup	Left	0.00199	CingulumPost	Right	0.00978
FrontalInfOper	Left	0.00238	TemporalMid	Left	0.01003
FrontalInfTri	Right	0.00247	Cerebelum9	Left	0.01181
TemporalSup	Right	0.00270	FrontalSupMedial	Right	0.01274
TemporalMid	Left	0.00302	FrontalInfOper	Right	0.01393
OccipitalSup	Left	0.00357	OccipitalMid	Left	0.01398
TemporalMid	Right	0.00357	SupraMarginal	Left	0.01573
Cerebelum8	Right	0.00422	FrontalMedOrb	Left	0.01595
Angular	Left	0.00477	Cerebelum8	Right	0.01782
Precentral	Right	0.00493	FrontalInfTri	Right	0.01858
CingulumMid	Right	0.00620	Calcarine	Right	0.01942
OccipitalMid	Left	0.00817	Cerebelum45	Left	0.02174

Figure S2. Continued.

Experiment – Right caudate					
Meditators vs Controls (Figure 3c)			Yogis vs Controls (Figure 3d)		
Node	Hemisphere	*p*	Node	Hemisphere	*p*
FrontalSupMedial	Right	0.00893	Insula	Right	0.02220
ParacentralLobule	Right	0.00911	ParacentralLobule	Left	0.02396
Vermis8	-	0.01001	FrontalSup	Left	0.02445
Calcarine	Right	0.01026	FrontalInfTri	Left	0.02946
Postcentral	Right	0.01197	Vermis45	-	0.03181
Cerebelum7b	Right	0.01240	CingulumAnt	Left	0.03371
FrontalMid	Left	0.01258	Vermis10	-	0.03453
Cuneus	Right	0.01340	ParietalSup	Left	0.03719
FrontalMedOrb	Left	0.01565	Hippocampus	Right	0.03990
CingulumPost	Left	0.01578	Lingual	Left	0.03991
TemporalPoleMid	Right	0.01677	OccipitalInf	Left	0.04025
OccipitalInf	Left	0.01699	TemporalSup	Right	0.04053
FrontalMid	Right	0.01711	FrontalSupOrb	Right	0.04277
TemporalPoleMid	Left	0.01712	Cerebelum7b	Right	0.04383
ParietalInf	Right	0.01712	Precentral	Left	0.04418
Lingual	Left	0.01777	FrontalMidOrb	Left	0.04453
FrontalMedOrb	Right	0.01901	Cerebelum9	Right	0.04483
FrontalMidOrb	Left	0.01989	Rectus	Right	0.04516
Putamen	Right	0.02015	Precentral	Right	0.04682
FrontalInfOper	Right	0.02103	Vermis8	-	0.04719
SupraMarginal	Left	0.02128			
Lingual	Right	0.02240			
Cerebelum9	Right	0.02459			
Insula	Right	0.02507			
Pallidum	Right	0.02507			
Vermis10	-	0.03039			
FrontalSupOrb	Right	0.03196			
Angular	Right	0.03624			
Cerebelum10	Right	0.03876			
FrontalSupOrb	Left	0.03922			
Postcentral	Left	0.04025			
Precuneus	Right	0.04053			
ParaHippocampal	Right	0.04063			
Vermis45	-	0.04145			
Precuneus	Left	0.04244			
OccipitalMid	Right	0.04543			
Cerebelum9	Left	0.04925			

Table S3. P-values of significant connections between the left and right caudate and all 115 other regions of the AAL atlas for meditators vs controls in the replication data-set, as displayed in Figure 3e and 3f.

Replication – Meditators vs Controls					
Left caudate (Figure 3e)			Right caudate (Figure 3f)		
Node	Hemisphere	*p*	Node	Hemisphere	*p*
FrontalInfOrb	Right	0.00009	FrontalInfOrb	Left	0.00214
FrontalSup	Left	0.00010	FrontalSupOrb	Left	0.00440
TemporalInf	Left	0.00032	FrontalSupMedial	Left	0.00708
TemporalMid	Left	0.00115	FrontalMidOrb	Right	0.00753
FrontalInfOrb	Left	0.00196	Heschl	Right	0.00868
Cerebelum9	Left	0.00251	FrontalInfTri	Left	0.00876
Lingual	Left	0.00324	FrontalInfOrb	Right	0.01082
Cerebelum7b	Left	0.00331	RolandicOper	Right	0.01337
SuppMotorArea	Right	0.00347	ParietalInf	Left	0.01375
TemporalPoleSup	Left	0.00388	FrontalMidOrb	Left	0.01485
Heschl	Right	0.00424	FrontalMid	Left	0.01536
CingulumAnt	Right	0.00481	ParietalInf	Right	0.01598
FrontalSupMedial	Right	0.00510	TemporalInf	Left	0.01621
TemporalPoleSup	Right	0.00534	TemporalPoleSup	Right	0.02351
CingulumMid	Right	0.00579	TemporalMid	Left	0.02386
FrontalMidOrb	Left	0.00631	Rectus	Left	0.02388
Angular	Right	0.00649	FrontalSupMedial	Right	0.02484
FrontalMidOrb	Right	0.00712	OccipitalSup	Right	0.02828
FrontalSupMedial	Left	0.00725	TemporalPoleSup	Left	0.03097
TemporalSup	Right	0.00787	Heschl	Left	0.03302
TemporalInf	Right	0.00825	Rectus	Right	0.03319
Fusiform	Right	0.00893	FrontalMid	Right	0.03397
TemporalMid	Right	0.00958	CingulumAnt	Left	0.03696
Precuneus	Right	0.00960	Cuneus	Right	0.03799
TemporalPoleMid	Left	0.00978	Olfactory	Left	0.03917
CerebelumCrus1	Left	0.01168	Cerebelum9	Left	0.03945
FrontalInfTri	Right	0.01252	Angular	Right	0.03965
OccipitalMid	Left	0.01298	TemporalSup	Left	0.04005
RolandicOper	Right	0.01376	TemporalPoleMid	Right	0.04414
FrontalMid	Left	0.01515	FrontalInfTri	Right	0.04950
ParacentralLobule	Left	0.01705			
CingulumAnt	Left	0.01894			
Cerebelum10	Left	0.02043			
Rectus	Right	0.02078			
CerebelumCrus2	Left	0.02489			
ParietalSup	Left	0.02524			

Table S3. Continued.

Replication – Meditators vs Controls					
Left caudate (Figure 3e)			Right caudate (Figure 3f)		
Node	Hemisphere	*p*	Node	Hemisphere	*p*
Cuneus	Left	0.02578			
Cerebelum45	Left	0.02677			
Precuneus	Left	0.02921			
Calcarine	Right	0.02932			
Cerebelum6	Right	0.03172			
Putamen	Left	0.03681			
RolandicOper	Left	0.03905			
CingulumPost	Left	0.03930			
CingulumMid	Left	0.03974			
TemporalSup	Left	0.04085			
ParacentralLobule	Right	0.04098			
Fusiform	Left	0.04235			
FrontalInfOper	Right	0.04289			
Amygdala	Left	0.04354			
FrontalInfTri	Left	0.04539			
Insula	Right	0.04677			
Cerebelum8	Left	0.04795			
Cuneus	Right	0.04806			
FrontalSupOrb	Left	0.04846			
SuppMotorArea	Left	0.04883			
FrontalMedOrb	Right	0.04910			
